# Cerebrospinal Fluid IgM and Oligoclonal IgG Bands in Multiple Sclerosis: A Meta-Analysis of Prevalence and Prognosis

**DOI:** 10.3390/brainsci11111444

**Published:** 2021-10-29

**Authors:** Mattia Fonderico, Emilio Portaccio, Lorenzo Razzolini, Luisa Pastò, Angelo Bellinvia, Ilaria Addazio, Matteo Betti, Maria Grazia Aprea, Clara Ballerini, Tiziana Biagioli, Maria Pia Amato

**Affiliations:** 1 Division Neurological Rehabilitation, Department of NEUROFARBA, University of Florence, 50139 Florence, Italy; mattia.fonderico1991@gmail.com (M.F.); dottlorenzo.razzolini@gmail.com (L.R.); luisa.pasto@yahoo.it (L.P.); angelobellinvia@outlook.it (A.B.); ilaria.addazio@gmail.com (I.A.); matteo_hhh@alice.it (M.B.); mariagrazia.aprea@unifi.it (M.G.A.); mariapia.amato@unifi.it (M.P.A.); 2 Department of Experimental and Clinical Medicine (DMSC), University of Florence, 50139 Florence, Italy; clara.ballerini@unifi.it; 3 Central Diagnostic Laboratory, Careggi University Hospital, 50134 Florence, Italy; biagiolit@aou-careggi.toscana.it; 4 IRCCS Fondazione Don Carlo Gnocchi, 50143 Florence, Italy

**Keywords:** multiple sclerosis, biomarker, intrathecal IgM synthesis, oligoclonal bands

## Abstract

The presence of intrathecal IgM synthesis (ITMS) has been associated with an aggressive multiple sclerosis (MS) clinical course. In the present systematic review, we aimed at assessing the prevalence of ITMS among different MS phenotypes. Moreover, we aimed at quantifying the risk of a second relapse in ITMS positive and oligoclonal IgG bands (OCGBs)-positive patients. We selected clinical studies reporting the ITMS prevalence assessed as oligoclonal IgM Bands (OCMBs), lipid-specific OCMBs (LS-OCMBs), and/or as an intrathecal IgM production > 0% (IgMLoc, Reiber formula). The overall prevalence of ITMS was higher in relapsing-remitting (RR) than clinically isolated syndrome (CIS) patients (40.1% versus 23.8%, *p* < 0.00001), while was in line with that detected in primary progressive MS (PPMS, 26.7%). Almost all patients (98%) with ITMS had also OCGBs. The risk of having a second relapse was higher in OCGBs positive patients (HR = 2.18, *p* = 0.007) but much higher in ITMS positive patients (HR = 3.62, *p* = 0.0005). This study revealed that the prevalence of ITMS is higher in RRMS patients. It suggests that the risk of having a second relapse, previously ascribed to OCGBs, may, to a certain extent, be related to the presence of intrathecal IgM.

## 1. Introduction

Accumulating evidence shows the positive long-term impact of early treatment with high efficacy therapies in multiple sclerosis (MS) patients [1,2]. However, such therapies could carry more serious adverse event profiles, underscoring the need for accurate, personalized prognostication in order to identify subjects with more aggressive disease who are most likely to benefit. Currently, clinical characteristics such as sex, age, relapse rate and recovery, the functional system involved at onset, and brain magnetic resonance imaging (MRI) parameters play the most important role [3,4]. Among biomarkers, the intrathecal synthesis of oligoclonal IgG bands (OCGBs) remains the most consistent laboratory abnormality in MS, present in the cerebrospinal fluid (CSF) of up to 95% of relapsing–remitting MS (RRMS) patients [5]. Furthermore, its presence demonstrates the dissemination in time of the disease and is associated with an increased likelihood of a second clinical relapse and confirmed disability progression [6]. However, the prognostic value of OCGBs is limited by their high prevalence, suggesting a milder disease course in the minority of patients where they are absent. 

On the other hand, an intrathecal IgM synthesis (ITMS) has been reported in 20-40% of MS patients [7,8,9]. It has been associated with a higher disability [10], higher risk of further relapses [11], shorter time to second relapse [7,8], conversion to secondary progressive MS (SPMS) [12], and a subset of primary progressive MS (PPMS) patients with more inflammatory phenotype [13]. However, other studies found no correlations [14,15,16] or contradicting results [17,18]. Moreover, while isoelectric focusing (IEF) on an agarose gel followed by immunoblotting/immunofixation for IgG has become the gold standard for OCGBs detection [19], no consensus exists regarding the best method to detect an ITMS. 

The presence of ITMS can be assessed quantitatively or qualitatively. Quantitative methods assess the serum and CSF immunoglobulin concentrations and through linear [20], exponential [21], or hyperbolic formulae [22,23] differently adjust for brain–barrier damages and age-related differences. Hyperbolic functions demonstrated better accuracy than linear functions and better estimated the intrathecally synthesized amount of IgM (IgMLoc) [22,24].

The detection of oligoclonal IgM bands (OCMBs) has technical difficulties compared to OCGBs. Very briefly, technical difficulties arose since IgM concentration in the CSF is 200 times less than IgG, immunoglobulins M are hydrophobic, and in vivo are aggregated in pentameric structure [22,25]. Notwithstanding, different groups demonstrated good interlaboratory reproducibility in the last years, suggesting different methods for introducing OCMBs in clinical practice [26,27,28].

In 2005, Villar LM and co-workers found that a considerable percentage of OCMBs recognize specific myelin lipidic antigens and named those bands lipid-specific OCMBs (LS-OCMBs) [29]. Subsequent studies found that patients with LS-OCMBs seemed to have an even more aggressive disease course [30,31] and a worse response to interferon-beta treatment [32].

The first aim of the present systematic review is to calculate the overall prevalence of ITMS assessed as LS-OCMBs, OCMBs, and intrathecal synthesized IgM > 0% assessed with Reiber formula [22] in different MS phenotypes. In addition, meta-regression analyses were performed to determine the hazard of having a subsequent clinical relapse in patients with ITMS and OCGBs.

## 2. Materials and Methods

We conducted a systematic literature review of the PubMed database using the following search strings: “Multiple Sclerosis” and (“immunoglobulin M” or “IgM” or “Intrathecal immunoglobulin synthesis”). Abstracts extraction was conducted as of 15 July 2021 and resulted in 1.140 hits, of which 324 were duplicates, and 816 were screened based on abstract and title. 

**Figure 1:**
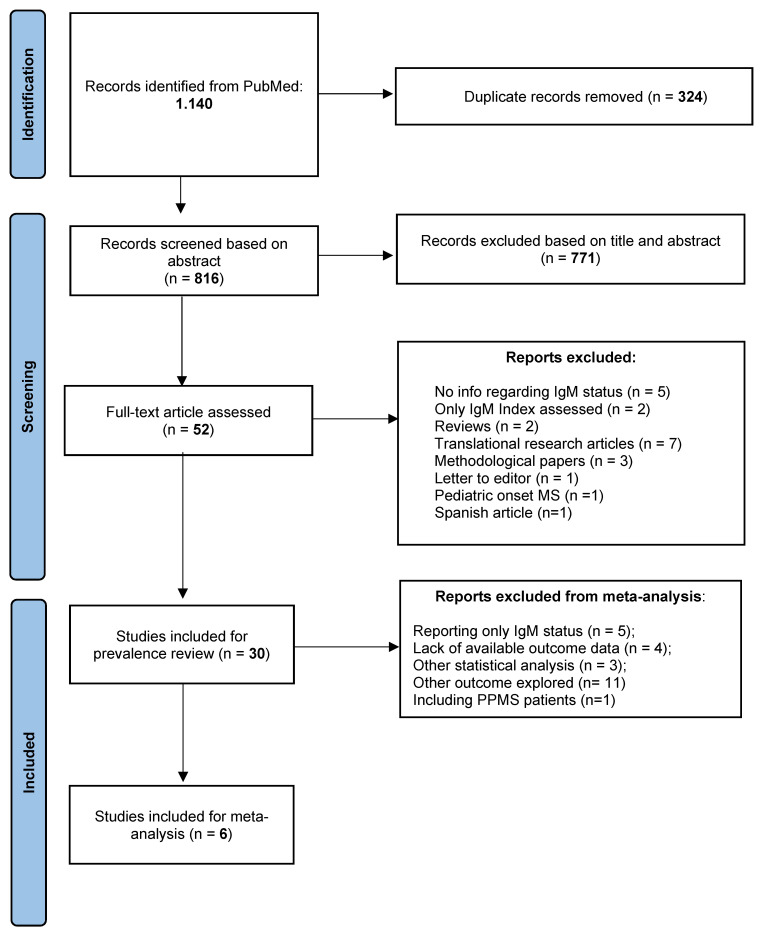
PRISMA flow chart representing literature review process.

For prevalence assessment, we selected longitudinal clinical cohort studies that reported the prevalence of ITMS assessed as LS-OCMBs, OCMBs, or IgMLoc (Reiber function) in adult MS patients (>18 years at disease onset) and in the English language. We included cross-sectional studies only if they recruited consecutive MS patients and not selected cohorts. If a study investigated more than one method for ITMS, different prevalence estimates were recorded. However, in the estimation of overall prevalence, we considered only the qualitative method (OCMBs or LS-OCMBs). Prevalence among different MS phenotypes (CIS, RRMS, or PPMS) was recorded only if the paper clearly stated the MS phenotype at the time of lumbar puncture (LP); otherwise, they were used for the overall analysis of prevalence estimation. By applying selection criteria, we assessed the full text of 52 papers, and 30 papers met the inclusion requirements for prevalence assessment (Figure 1). 

For the quantitative synthesis, we further retained prospective longitudinal studies that assessed the hazard of having a second clinical relapse or converting to clinically definite MS (if the conversion was assessed as a new clinical event). We retained only papers that clearly stated follow-up duration, IgM and IgG status, phenotype at baseline, univariate hazard ratios (HRs), and 95% confidence intervals (CI). Papers that showed Kaplan–Meier curves without reporting the number at risk were excluded. By applying selection criteria, six studies were included in the meta-analysis for the risk of a second relapse (Figure 1).

### Statistical Analysis

The prevalence of ITMS was calculated using standard formulae. For differences in prevalence among groups, a chi-square test was used. 

Data regarding inclusion criteria, duration of follow-up, IgM status, age at disease onset, MS diagnostic criteria, phenotype at the time of LP, and results were extracted from each study (Supplementary Table S1). For phenotype identification (CIS, RRMS, and PPMS), we considered only the studies that specified disease course at the time of LP. In addition, as a sensitivity analysis, we excluded studies that referred to the 2017 McDonald criteria.

The univariate hazard ratios (HRs) for the risk of a second clinical relapse was extracted from the six selected studies (Figure 1). Between-study heterogeneity was assessed using Cochran’s Q, chi-square, and I^2^ test. A random-effects model was applied unless I^2^ was <25%; otherwise, a fixed model was used. Publication bias was assessed using visual inspection of funnel plots (Supplementary Figure S1). All study-specific estimates were combined using inverse variance-weighted averages of logarithmic HRs in both random- and fixed-effects models. We performed meta-analysis and plots using Review Manager Web (RevMan Web) version 5.4 available at www.revman.cochrane.org (accessed on 1 September 2021). 

## 3. Results

Following the initial screening, 816 unique papers were screened, and 52 papers were fully assessed, of whom 30 met inclusion criteria for prevalence assessment and 6 for quantitative synthesis (Figure 1). A summary of findings for each study is available as supplementary material (Supplementary Table S1). 

### 3.1. Prevalence of ITMS in CIS, RRMS, and PPMS Patients

For prevalence estimation, we retained 30 studies (14 retrospective, 14 prospective, and 2 cross-sectionals) which included a total of 5000 patients: 1881 CIS, 1583 RRMS, 467 PPMS, and 1069 with no clearly stated phenotype at the time of LP (CIS or RRMS).

The overall prevalence of an ITMS was 29.0% (1450/5000), and it was 30.5% (299/981), 34.8% (673/1932), and 22.8% (583/2562) when detected as LS-OCMBs, OCMBs, and IgMLoc, respectively.

The overall prevalence of an ITMS in CIS patients was 23.8% and was significantly lower than the overall prevalence assessed in RRMS patients (40.1%, *p* < 0.00001). The prevalence of intrathecal IgM was lower in CIS than in RRMS patients also when assessed as LS-OCMBs (23.7% versus 38.9%, *p* < 0.00001), OCMBs (33.0% versus 48.5%, *p* < 0.00001), and IgMLoc (18.8% versus 31.3%, *p* < 0.00001). The prevalence of IgG OCBs was lower in CIS than in RRMS (80.5% versus 91.0%, *p* < 0.05). We excluded the studies that referred to the 2017 revision of McDonald criteria [8,27,33,34,35,36] as sensitivity analyses. The results did not change (CIS versus RRMS patients, 23.9% 338/1412 vs. 42.3% 412/973, *p* < 0.00001). In PPMS, the overall prevalence of an ITMS was 27%, in line with that detected in relapsing patients.

Seventeen studies reported the prevalence of OCGBs among patients with ITMS. In the studies providing this information, almost all (97.6%, 528/541) MS patients with ITMS also had oligoclonal IgG bands. 

### 3.2. Relationship between IgM and IgG Status and Second Relapse

**Figure 2:**
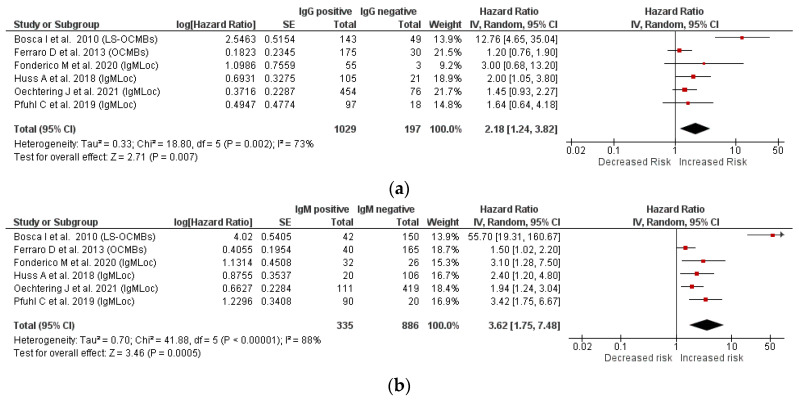
Forest plot for the risk of a second clinical relapse in patients with IgG oligoclonal bands (**a**) and patients with intrathecal IgM synthesis (**b**).

Six longitudinal studies (follow-up range: 2–9.6 years) that assessed both IgM and IgG status and recruited a total of 1221 CIS/early RRMS patients were included in the meta-analysis. Four studies assessed the IgM status as IgMLoc, one study as LS-OCMBs, and one as OCMBs. The pooled analysis confirmed that the presence of OCGBs is a risk factor for a second clinical relapse (HR = 2.18, 95% CI 1.24–3.82, I^2^ = 73%, *p* = 0.007, Figure 2a). The risk of a second relapse was much greater in patients with ITMS (HR = 3.62, 95% CI 1.75–7.48, I^2^ = 88%, *p* = 0.0005, Figure 2b). As a sensitivity analysis, we included only the four studies with the same method of ITMS detection (IgMLoc). In the subgroup analysis (Supplementary Figure S1), the between-study heterogeneity decreases to less than 25% and the risk of having a second relapse was confirmed to be higher in IgM positive (HR = 2.41, 95% CI 1.78–3.28, I^2^ = 0%, *p* < 0.00001) than OCGBs positive patients (HR = 1.67, 95% CI 1.19–2.33, I^2^ = 0%, *p* = 0.003).

## 4. Discussion

By pooling a large number of studies with information regarding the IgM status, we found that in MS patients, the overall prevalence of an ITMS was 29.0%, and it was higher in RRMS (40.1%) than in CIS patients (23.8%, *p* < 0.0001). Almost all patients with OCGBs also had a positive IgM status (98%). By meta-regression analysis, we found that patients with ITMS were at higher risk of having a second clinical relapse (HR = 3.62, *p* = 0.0005), a risk that appeared to be higher than that conferred by OCGBs (HR = 2.18, *p* = 0.007). 

Whether intrathecal immunoglobulins are pathogenic or represent markers of active CNS inflammation is still under debate; the same applies to the immunopathological mechanisms of the prognostic role of IgG and, in particular, IgM CSF status. Pathological studies described four different patterns of demyelination [37]. These patterns are stable within individual patients [38], and only pattern II shows antibody-mediated demyelination [37,39]. Both IgG and IgM localized on oligodendrocytes and axons, and they co-localized with complement and foamy macrophages [40]. With their multimeric structure, IgM antibodies are the strongest complement activator, which can cause more pronounced demyelination and axonal damage [40]. A single bound IgM pentamer can trigger the classical pathway of complement activation and lyse a red blood cell. Conversely, approximately a thousand IgG molecules are required to accomplish the same result [41]. Moreover, in animal models, IgM directed against glycolipids induced CNS demyelination and prevented remyelination [42]. 

From the immunological point of view, intrathecal IgM in MS patients presents some peculiar features. First, in contrast to IgM chains of peripheral blood, intrathecal IgM is commonly characterized by the lack of switch from IgM to IgG class and by a high degree of somatic hypermutation (SHM) [43,44]. Secondly, ITMS persists as a characteristic feature of MS [43,45]. Moreover, different from intrathecal IgG restricted bands, which mainly target non-self-antigens (measles, rubella, mumps, and many others [46]), a subset of intrathecal IgM target myelin lipids (in most cases phosphatidylcholine, followed by phosphatidylinositol, gangliosides, and sulphatides [29,31,47]). It remains unclear why and how the CSF-resident IgM memory B cells are triggered to initiate SHM and to produce intrathecal IgM [42], but the persistence of ITMS indicates that it is not a primary immune response yet it is a persistent one. A recent study reported the findings in the CSF of 108 patients with myelin oligodendrocyte glycoprotein (MOG) antibody [48]. Interestingly, most anti-MOG patients lacked OCGBs, and 5 out of 13 patients with ITMS (assessed as IgMLoc) had only evidence of IgM but not IgG synthesis. Moreover, ITMS was observed only during acute relapses but not during the remission phase [48], which is in striking contrast with the temporal invariance observed in MS, and it suggests different pathogenesis of the two disorders. 

From the clinical point of view, emerging evidence shows that disease-modifying treatments (DMTs) may alter intrathecal immunoglobulins production and that intrathecal immunoglobulins may modulate DMT response. For example, natalizumab decreased serum IgM and IgG levels but, in CSF, only IgG indices [49]. Furthermore, it has been demonstrated that autologous hematopoietic stem cell transplantation (aHSCT) lowered intrathecal immunoglobulin indices and suppressed both IgG and IgM oligoclonal bands, thus challenging the notion that OCBs are unaffected by therapeutic intervention in MS [50]. Additionally, the response to interferon beta seems to be lower in patients with LS-OCMBs [32].

On the whole, our finding that the prevalence of ITMS is higher in RRMS than CIS patients is consistent with the pathological and immunological evidence exposed above. If an intrathecal IgM synthesis predicts future relapse activity and is persistent within the CSF, it is reasonable to believe that the proportion of IgM positive patients increases among patients who experienced more than one relapse. In this study, the prevalence of ITMS was persistently higher in RRMS than CIS patients for all three methods for ITMS detection (LS-OCMBs, OCMBs, and IgMLoc). It should be noted that the definition of CIS changed with the 2017 revision of the McDonald criteria, as it permits to make the diagnosis of definite MS at the first demyelinating event after having excluded other explanations [51]. However, in our pooled analysis, the proportion of patients with OCGBs among CIS (80%) and RRMS patients (91%) is consistent and comparable with that observed in other large CIS cohorts (72-85%) [52,53,54] and RRMS meta-analysis studies (88%) [5]. Moreover, we confirmed this result excluding the studies referring to the 2017 revision of the McDonald criteria.

We also found that almost all (98%) patients who presented intrathecal IgM also had restricted IgG oligoclonal bands. Moreover, our study confirmed that OCGBs are a risk factor for a second clinical relapse (HR = 2.2), in line with that found in a large and well-documented CIS cohort (HR = 2.8) [6]. It should also be noticed that in our study, we chose as a clinical outcome the hazard of having a second clinical relapse. While the reference class for OCGBs are the patients without OCGBs, the reference class for the patients with ITMS comprises both OCGBs negative and positive patients, for whom the risk of a second relapse has been demonstrated to be higher. Thus, the risk of having a second clinical relapse in patients with ITMS compared to OCGBs could be even higher. Altogether, these results support the hypothesis that the risk of conversion to MS, previously ascribed to OCGBs, may to a certain extent be related to the presence of an ITMS.

A major debate regards the best method for detecting the ITMS. A recent study confirmed that the quantitative method described by Reiber is specific (97.6%) but with low sensitivity (23%) compared to qualitative detection [27]. Our analysis confirms this finding, as the prevalence of ITMS was consistently higher when detected with qualitative methods (OCMBs or LS-OCMBs). Quantitative methods have the advantages to be low-cost, easy to interpret, available in most laboratories, and thus potentially includible in routine clinical practice and clinical trials. On the other hand, qualitative methods are more accurate but also more laborious. The pH range required for IEF of IgM ranges from 5 to 8 (instead of the pH = 3–10 required for IgG [27]), and the circulating IgM pentamers can migrate through the gel on IEF only after they have been reduced, with the risk of arbitrary reassociation and false-positive and -negative results [46]. Moreover, the cut-off normally used is the presence of two or more bands in the CSF not present in the paired serum sample [26], but a four-band cut-off has also been proposed [27]. Despite these limitations, semi-automated methods have been developed and could be included in clinical practice in the future [28]. A recent study by Oechtering J et al. found that among IgMLoc (Reiber formula) positive patients, the ones who had a more pronounced intrathecal synthesis (>median) had a shorter time to first relapse in comparison to patients who had a less pronounced synthesis (IgMLoc < median [34]). If this result is confirmed in other future studies, we might be interested in knowing not only the presence and the type of immunoglobulin but also its quantitative amount.

## 5. Limitations

In interpreting our results, we should note that we included only univariate analyses, which did not consider important covariates such as clinical prognostic factors and the administration of DMT. Moreover, we did not include some studies that reported conflicting results because of different study designs and statistical methods. However, some of these studies evaluated relapses by telephone interview [14,17], opening the possibility for recall bias. Furthermore, the possibility of publication bias cannot be ruled out. Notwithstanding, the prevalence of OCGBs in CIS and RRMS patients, as well as the risk of a second relapse that we found for OCGBs, appear to be strictly in line with the available literature.

## 6. Conclusions

A plethora of new DMTs is now available with a wide range of activity and associated risks, and biomarkers that predict future relapse activity are needed to improve the benefit–risk balance. By pooling a large number of studies, we reported the overall prevalence of ITMS among 5000 MS patients and found that ITMS is higher in RRMS than CIS patients. By selecting comparable studies with both IgM and IgG status, our findings suggest that the risk of having a second relapse, previously ascribed to OCGBs, may, to a certain extent, be related to the presence of intrathecal IgM. Therefore, ITMS can represent a reliable and accurate prognostic marker to be incorporated in the therapeutic decision-making process. Further studies are needed to clarify the relationship between the presence of intrathecal immunoglobulins and MS clinical course.

## Data Availability

Anonymized data, not published in the article, will be shared on reasonable request from a qualified investigator.

## Supplementary Materials

The following are available online at https://www.mdpi.com/article/10.3390/brainsci11111444/s1, Figure S1: Forest plot for the risk of a second clinical relapse. Only studies with the same method of intrathecal IgM detection are analyzed; Table S1: Summary of findings.

## Abbreviations

CIS—clinically isolated syndrome; CSF—cerebrospinal fluid; DE—demyelinating event; DMD(s)—disease-modifying drugs; EDSS—Expanded Disability Status Scale; IgMLoc—intrathecal synthesized IgM > 0% assessed with Reiber formula; ITMS—intrathecal IgM synthesis; LP—lumbar puncture; LS-OCMBs—lipid-specific OCMBs; OCGBs—olicoclonal IgG bands; OCMBs—oligoclonal IgM bands.
