# Supplementary material for: Cerebrospinal Fluid IgM and Oligoclonal IgG Bands in Multiple Sclerosis: A Meta-Analysis of Prevalence and Prognosis
Source: Brain Sci. 2021 Oct 29;11(11):1444. doi: 10.3390/brainsci11111444 (PMC8615995; doi:10.3390/brainsci11111444)
Supplement: Supplementary file 1 [file brainsci-11-01444-s001.zip › brainsci-1429173-supplementary.pdf]

Reference	Study design	Follow-up	ITMS detection	Cases and sample size (mean age)	McDonald Criteria	Main clinical Outcome(s)	Results
Abdelhak A, et al. *CSF profile in primary progressive multiple sclerosis: Re-exploring the basics.* PLoS One 2017	Retrospective cohort study	27 mo	IgMLoc	176 PPMS (47)	2010	Yearly progression rate	In patients with ITMS, a negative correlation was found between IgMloc and yearly progression rate (ρ = − 0.4, p = 0.03, n = 28).
Berek K, et al. *Cerebrospinal Fluid Findings in 541 patients with Clinically Isolated Syndrome and Multiple Sclerosis: A Monocentric Study*. Front Immunol 2021	Retrospective cohort study	9 Yr	IgMLoc	80 CIS, 240 McDonald MS**, 155 RRMS, 43 PPMS (34)	2017	To assess routine CSF parameters in MS patients and the frequency of pathologic findings by applying novel reference limits.	In patients with LP at the first relapse, elevation of WBC count [odds ratio (OR) 2.9, 5% CI: 1.0 – 8.2, p=0.049] as well as the presence of monocytes/macrophages in CSF cytology (OR 7.3, 95% CI: 1 .4 – 38.2, p=0.018) were predictive for the occurrence of a second clinical relapse during follow-up. Blood-CSF-barrier dysfunction and intrathecal IgA/IgM synthesis are less frequent when the novel reference limits are applied.
Bosca I, et al. *Response to interferon in multiple sclerosis is related to lipid-specific oligoclonal IgM bands.* Mult Scler 2010	Prospective cohort study	37 mo	LS-OCMBs	102 RRMS (33)	2005	Time to first relapse after IFNβ, relapse rate, relapse rate reduction, proportion of relapse-free pts	LS-OCMBs increased the risk of second relapse (HR = 2,0, 95% CI 1,2-3,5, p = 0,01). LS-OCMBs patients showed lower reduction in relapse rate (51.8% versus 80.8%; p<0.0001), higher relapse rate in the first year (0.8 versus 0.2; p < 0.001), and lower proportion of relapse-free patients (25% versus 61.3%; p < 0.003).
Boscá I, et al. *The risk of relapse after a clinically isolated syndrome is related to the pattern of oligoclonal bands*. J Neuroimmunol 2010	Prospective cohort study	6,7 Yr	LS-OCMBs	192 CIS (32)	NS	Time to second relapse (determining a CDMS)	Kaplan–Meier survival analysis showed that the median time from the onset of CIS to the second relapse was 0.7 years when both OCGBs and LS-OCMBs were present, 3.3 years when OCGBs were present but LS-OCMBs were absent (95% CI 2.6 to 4.0) and 9.9 years when both types of oligoclonal bands were absent. Kaplan–Meier log rank was 124.6 with p<0.001. Considering only patients with OCGBs (n=143), the risk of having a new relapse was significantly higher for patients with additional LS-OCMBs in the CSF (HR 4.4, 95%CI 2.8-6.9, p < 0.0001)
Calabrese M, et al. *The association of intrathecal immunoglobulin synthesis and cortical lesions predicts disease activity in clinically isolated syndrome and early relapsing-remitting multiple sclerosis.* Mult Scler J 2012	Retrospective cohort study	3 Yr	IgM Loc	46 CIS, 40 RRMS (32)	2005	Conversion to CDMS, number of new relapses, EDSS worsening, new T2 white matter lesions, new cortical lesions	Mean number of CLs was higher in IgMloc positive (7.24.5, range: 0–14) than negative patients (2.02.5, range: 0–6 p <0.001). Risk of conversion to CDMS was higher for IgMloc positive patients (RR 1.8; 95% CI 1.1–2.8; p < 0.05). IgMloc predicted new clinical or MRI disease activity (RR 1.5; 95% CI 1.2–1.9; p < 0.05).

Capuano R, et al. *Oligoclonal IgM bands in the cerebrospinal fluid of patients with relapsing MS to inform long-term MS disability*. Mult Scler J 2021	Prospective cohort study	9,6 Yr	OCMBs / LS-OCMBs	89 RRMS (33)	2010/2017	Time to EDSS 3.0 / EDSS 4.0	OCMBs were associated with a 33% increase in the annualized relapse rate (ARR; p = 0.06), higher odds for high-efficacy DMDs use (OR = 4.8; 95% CI = (1.5, 16.1)), thinner pRNFL (β = -4.4; 95% CI = (-8.6, -0.2)) and GCIPL (β = -2.9; 95% CI = (-5.9, +0.05)), and higher hazard ratios of EDSS ⩾ 3.0 (HR = 4.4; 95% CI = (1.6, 11.8)) and EDSS ⩾ 4.0 (HR = 5.4; 95% CI = (1.1, 27.1)).
Casanova B, et al. *Different clinical response to interferon beta and glatiramer acetate related to the presence of oligoclonal IgM bands in CSF in multiple sclerosis patients*. Neurol Sci 2018	Retrospective cohort study	41 Mo	OCMBs	256 RRMS (32)	2005	Time to second relapse after having started IFN or GA	54.7% of patients remained free from further relapses (RF). The proportion of RF patients was higher in the GA group than in the IFNβ group (72.2 vs. 40.4%, p < 0.001). The IFNβ patients with OCMB presented the poorest response, 31.3% RF vs. 48.1% in IFNβ without OCMB (p = 0.03). The highest risk of relapse after treatment was for patients with OCMB in CSF and receiving IFNβ treatment, followed by patients without OCMB and receiving IFNβ treatment (p = 0.0001).
Ferraro D, et al. *Cerebrospinal fluid oligoclonal IgM bands predict early conversion to clinically definite multiple sclerosis in patients with Clinically Isolated Syndrome.* J Neuroimmunol 2013	Retrospective cohort study	102 Mo	OCMBs	205 CIS (32)	NR (First demyelinating event)	Time to second relapse	At survival analysis, a lower age at onset, an onset with optic neuritis and the presence of OCMBs increased the risk of a second relapse. Only the presence of OCMBs predicted a relapse within one year (1.5 (1.0-2.2 p = 0.043)). Median time to second relapse: 20 months in OCMB+ patients, 35 months in OCMB- patients.
Ferraro D, et al. *Cerebrospinal fluid CXCL13 in clinically isolated syndrome patients: Association with oligoclonal IgM bands and prediction of multiple sclerosis diagnosis*. J Neuroimmunol 2015	Retrospective cohort study	41 mo	OCMBs	110 CIS (35)	2010	Time to CDMS (clinical or radiological)	An elevated CSF cell count (HR: 1.04; p = 0.006), a positive baseline MRI (defined by the presence of at least one lesion on T2-weighted images) (HR: 5; p = 0.029), a positive baseline spinal cord MRI (HR: 1.7; p = 0.017) and CSF CXCL13 levels ≥ 15.4 pg/ml increased the risk of MS diagnosis (HR: 3; p = 0.001). Rate of conversion to CDMS between OCMBs positive and negative patients was similar (OR = 0.6, p = 0.275).
Fonderico M. et al. *Prognostic role of intrathecal IgM synthesis in multiple sclerosis: Results from a clinical series.* Mult Scler J 2020	Prospectively acquired data	2.9 Yr	IgM Loc	6 CIS, 52 RRMS and 10 PPMS (37)	2017	Time to second relapse	RRMS patients with ITMS were at higher risk of experiencing a second clinical relapse (adjusted hazard ratio (aHR) = 6.3, 95% confidence interval (CI) = 2.1–18.4, p = 0.001) and had a high T1 lesion load (p < 0.05).
Frau J, et al. *Intrathecal oligoclonal bands synthesis in multiple sclerosis: is it always a prognostic factor?* J Neurol 2018	Retrospective cohort study	6 Yr	OCMBs	479 RRMS and 24 PPMS (38)	2010	Time to EDSS 3.0 and EDSS 4.0	Cox regression showed that higher age at onset (HR = 1,04, 95% CI 1,02-1,07, p < 0,005) and clinical course PP (HR = 2,8, 95% CI 1,5-5,2, p = 0,001) conferred an increased risk of reaching EDSS 3,0. The presence of

							OCMBs and OCGBs did not increase the risk of EDSS 3.0/4.0.
García-Barragán N, et al. *Multiple sclerosis patients with anti-lipid oligoclonal IgM show early favourable response to immunomodulatory treatment*. Eur J Neurol 2009	Prospective cohort study	70, 5 Mo	LS-OCMBs	75 CIS (32)	NR (first demyelinating event)	EDSS progression and percentage of treated patients during follow-up.	Immunomodulatory treatments (IT) similarly reduced relapse rate in both treated groups (P < 0.0001) and reduced EDSS progression in LS-OCMBs+ patients, whose EDSS score had significantly increased before treatment.
Gasperi C, et al. *Association of Intrathecal Immunoglobulin G Synthesis with Disability Worsening in Multiple Sclerosis*. JAMA Neurol 2019	Prospectively acquired data	4 Yr	IgM Loc	319 CIS and 354 RRMS (34)	2005	Risk of EDSS worsening after 4 years (see main Text)	Intrathecal IgG synthesis was associated with a higher risk of EDSS worsening after 4 years (odds ratio, 2.02 [95% CI, 1.15-3.58]; P = .01), independent of the occurrence of relapses and disease-modifying therapy. No association of other routine cerebrospinal fluid parameters nor IgM status was found with EDSS worsening.
Gil-Perotin S, et al. *Combined Cerebrospinal Fluid Neurofilament Light Chain Protein and Chitinase-3 Like-1 Levels in Defining Disease Course and Prognosis in Multiple Sclerosis*. Front Neurol 2019	Cross sectional cohort	NR	OCMBs	99 RRMS and 58 PPMS (28 and 41)	2017	To investigate CSF Neurofilament Light Chain Protein and Chitinase-3 Like-1 levels in MS patients to evaluate the informative and prognostic potential of the individual and combined measures	In RRMS patients, CSF NFL and CHI3L1 levels correlated with each other (r = 0.58), and with OCMBs (p = 0.02 and p = 0.004, respectively). In addition, CSF CHI3L1 concentration was a predictor for 1-point EDSS worsening {HR = 2.99 [95% CI (1.27, 7.07)]} and progression during follow-up {HR = 18 [95% CI (2.31, 141.3)]}.
Huss A, et al. *Intrathecal immunoglobulin M production: A promising high-risk marker in clinically isolated syndrome patients*. Ann Neurol 2018	Prospectively acquired data	88 Mo	IgM Loc	126 CIS (34)	2010	Conversion to CDMS	The median conversion time for patients with ITMS was 10 months (95 % CI 3-17), 38 months (95 % CI 27-49), for patients without ITMS. The presence of IgM increased the risk of conversion to CDMS (HR = 2.4 95 % CI 1.2-4.6, p=0.002).
Klein A, et al. *CSF parameters associated with early MRI activity in patients with MS*. Neurol Neuroimmunol NeuroInflammation 2019	Retrospective cohort study	12 Mo	IgM Loc	149 CIS/Early RRMS	NR (first demyelinating event)	Risk of MRI progression	Of the 20 patients with ITMS, 15 showed cMRI activity in the follow-up scan (OR 2.861, CI 0.993–9.572; p = 0.064). OCGBs were positively associated with the occurrence of cMRI activity after 12 months in the entire cohort (OR 1.855, CI 0.824–4.279; p = 0.139) and in particular in DMT− patients (9.327, CI 2.296–37.890; p = 0.002) but not DMT+ patients (OR 0.714, CI 0.260–1.960; p = 0.513)
Magraner MJ, et al. *Brain atrophy and lesion load are related to CSF lipid-specific IgM oligoclonal bands in clinically isolated syndromes*. Neuroradiology 2012	Prospective cohort study	2 Yr	LS-OCMBs	24 CIS (36)	2005	T2 lesion volume (T2LV) and brain atrophy	T2LV at the end of follow-up (year 2) was threefold higher in patients with LS-OCMB (3.95 cm3 vs. 1.36 cm3, p= 0.001). Brain atrophy was also higher in patients with LS-

							OCMB (BPF, 0.73 in LS-OCMB+ patients vs. 0.76 in negative ones, p=0.03).
Mandrioli J, et al. *A multifactorial prognostic index in multiple sclerosis: Cerebrospinal fluid IgM oligoclonal bands and clinical features to predict the evolution of the disease*. J Neurol 2008	Retrospective cohort study	10 Yr	OCMBs	64 RRMS and a validation group of 65 RRMS (28)	2005	Time to EDSS 3.0 and EDSS 4.0	The probability of reaching an EDSS score of 3.0 or 4.0 was significantly influenced by the presence of OCMBs (p < 0.01 and p < 0.01, log-rank test) and by the symptoms at onset (p = 0.04 and p = 0.03, log-rank test). The presence of OCMBs and motor symptoms at onset significantly increased the risk of reaching EDSS 4.0 (HR = 5.65, CI = 1.86–17.16 and HR = 4.09, CI = 1.13–14.79, respectively)
Monreal E, et al. *Predicting Aggressive Multiple Sclerosis with Intrathecal IgM Synthesis Among Patients with a Clinically Isolated Syndrome*. Neurol Neuroimmunol Neuroinflammation 2021	Prospectively acquired data	12, 9 Yr	LS-OCMBs / OCMBs / IgMLoc	193 CIS (31)	2017	Risk of second relapse, EDSS 4.0, EDSS 6.0 and risk of conversion to SPMS.	Both OCMB and LS-OCMB were significantly associated with a higher risk of a second relapse (aHR 2.11, 95% CI 1.51–2.96, p < 0.001; and aHR 2.50, 95% CI 1.72–3.64, p < 0.001, respectively). The risk of EDSS 4.0 at the end of follow up was highest among LS-OCMBs patients (aHR 2.96, 95% CI 1.54–5.71; p = 0.001).
Oechtering J, et al. *Intrathecal Immunoglobulin M Synthesis is an Independent Biomarker for Higher Disease Activity and Severity in Multiple Sclerosis.* Ann Neurol 2021	Prospectively acquired data	5,1 Yr	IgM Loc	530 CIS/RRMS (33)	2017	Risk and time to second relapse and disability accrual (MS severity score)	OCGB+/IgGIF+/IgMIF+ patients had a 94% higher risk of experiencing a second relapse (HR = 1.94, p< 0.01). OCGB+/IgGIF+/IgMIF+ patients had higher sNfLZ-scores when compared with patients without intrathecal immunoglobulin synthesis (OCGB/IgGIF/IgMIF; estimate 0.41 [CI0.04, 0.78],p=0.032). OCGB+/IgGIF+/IgMIF+ patients had, on average, 2.5 times more T2w lesions compared with patients without intrathecal immunoglobulin (RR = 2.53[CI 1.63, 3.93],p< 0.01).
Pfuhl C, et al. *Intrathecal IgM production is a strong risk factor for early conversion to multiple sclerosis.* Neurology 2019	Prospective cohort study	2 Yr	IgM Loc	115 CIS and 35 early RRMS (33)	2010	Clinical (second relapse) or radiologic (new T2 or gadolinium enhancing lesions) conversion to MS	ITMS in patients with CIS was strongly associated with conversion to MS according to the McDonald 2010 criteria (HR = 3.05 [1.45–6.44], p = 0.003).
Schneider R, et al. *Intrathecal IgM-synthesis does not correlate with the risk of relapse in patients with a primary demyelinating event*. Eur J Neurol 2007	Retrospective cohort study	60 mo	OCMBs	42 CIS (31)	2001	Risk of second relapse and conversion to CDMS	There was no correlation between the presence of OCMBs, the number of bands or the IgM-Index and the risk of a relapse during the follow-up in the cohort studied.
Schwenkenbecher P, et al. *Reiber's diagram for kappa free light chains: The new standard for assessing intrathecal synthesis?* Diagnostics 2019	Retrospective cohort study	50 Mo	IgM Loc	68 CIS and 100 RRMS (30-36)	2017	-	Reiber's Kappa Free Light Chain diagram shows a great diagnostic performance to detect an intrathecal KFLC production in patients with MS.
Sola P, et al. *Primary progressive versus relapsing-onset multiple sclerosis: Presence and prognostic value of cerebrospinal fluid oligoclonal IgM*. Mult Scler J 2011	Retrospective cohort study	9 Yr	OCMBs	45 PPMS and 104 RRMS (48)	2005	Time to EDSS 3.0 and EDSS 4.0	Considering the relapsing-onset group (RR and SPMS), the presence of OCMBs significantly increased the risk of

							progression to an EDSS of 4.0 (HR=2.35, CI=1.14–4.83, p = 0.02)
Tejeda-Velarde A, et al. *Clinical usefulness of prognostic biomarkers in optic neuritis*. Eur J Neurol 2018	Prospective cohort study	46, 4 Mo	LS-OCMBs	68 Optic neuritis CIS (32)	2010	Risk of conversion to CDMS	LS-OCMB increased the risk of conversion to CDMS (HR = 116.6, CI = 13.4-1013, p = 0.002).
Thangarajh M, et al. *Lipid-specific immunoglobulin M in CSF predicts adverse long-term outcome in multiple sclerosis*. Mult Scler 2008	Retrospective cohort study	11 Yr	LS-OCMBs	81 RRMS (31)	2001	Risk of EDSS 4.0 and conversion to SPMS	The median time to reach an EDSS score of 4.0 was 14 years in LS-OCMB group and 24 years in the negative group (P = 0.002; hazard ratio = 3.7 [95% CI, 2.3–17.9]).
Villar LM, et al. *Immunoglobulin M oligoclonal bands: Biomarker of targetable inflammation in primary progressive multiple sclerosis*. Ann Neurol 2014	Cross-sectional cohort / Olympus PPMS cohort / validation cohort (23)	7,4 yr	OCMBs	80 PPMS in cross-sectional, 23 PPMS in Olympus and 67 in the international cohort (49)	2001 and 2005	Average MSSS score, prevalence of enhancing lesions, average number of enhancing lesions.	OCMB+ patients had, on average, a higher MSSS score (8.06 vs 6.47, p = 0.003), a higher average number of Gd enhancing lesions at baseline (p=0.0005) and a higher average number of new Gd enhancing lesions (1.09±0.35 for OCMB+ patients versus 0.17±0.14 for OCMB- patients).
Villar LM, et al. *Intrathecal IgM synthesis predicts the onset of new relapses and a worse disease course in MS*. Neurology 2002	Prospective cohort study	21, 4 mo	OCMBs	21 RRMS 1 PPMS (28)	Poser's criteria	Number of relapses; changes in the EDSS score	Patients with OCMBs had more relapses (mean, 2.0) than those without (mean 0.58, p = 0.02). At the end of the study, patients with OCMBs had higher EDSS scores (mean, 1.70) than those without (mean, 0.79, p = 0.02).
Villar LM, et al. *Intrathecal IgM synthesis is a prognostic factor in multiple sclerosis*. Ann Neurol 2003	Retrospective cohort study	11 Yr	OCMBs	29 RRMS (29)	Poser's critera	Probability of EDDS 6.0, EDSS at follow up, probability of conversion to SPMS, progression index, benign MS	During the follow-up, 70.8% of patients with ITMS converted to SPMS. None of the patients without ITMS did. At the end of the study, 63.6% of patients with ITMS had reached EDSS 6.0, whereas none of the patients lacking ITMS reached values above EDSS 3.0.
Zeman D, et al. *Cerebrospinal fluid oligoclonal IgM test in routine practice: Comparison with quantitative assessment of intrathecal IgM synthesis*. Clin Chim Acta 2020	Prospective cohort study	NR	OCMBs	4 CIS, 31 RRMS, 380 other diseases (NR)	2017	To explore the relationship between different formulas and results of ITMS, and to assess the frequency of ITMS in an unselected series of samples.	Formulas for intrathecal IgM synthesis are specific (95%-97.6%) but insensitive (15%-26%) compared to oligoclonal IgM Bands. Authors propose to evaluate samples with 2 or 3 extra-CSF IgM bands as borderline and only samples with 4 or more as definitely positive

Supplementary Table S1. Summary of findings.
* Mean or median
**Patients with one clinical relapse fulfilling 2017 McDonald criteria

Abbreviations: **CDMS** = Clinically Definite MS; **CI** = Confidence Interval; **CIS** = Clinically Isolated Syndrome; **CHI3L1** = Chitinase-3-like protein 1; **CL** = Cortical Lesions; **CSF** = CerebroSpinal Fluid; **DE** = Demyelinating Event; **DMD(s)** = Disease Modifying Drugs; **EDSS** = Expanded Disability Status Scale; **GA** = Glatiramer Acetate; **GCIPL** = Ganglion Cell plus Inner Plexiform Layer; **HR** = Hazard Ratio; **IgMLoc** = Intrathecal synthesized IgM > 0% assessed with Reiber formula; **IF** = Intrathecal Fraction; **IFN** = Interferon; **ITMS** = Intrathecal IgM synthesis; **KFLC** = Kappa Free Light Chain; **LP** = Lumbar Puncture; **LS-OCMBs** = Lipid-Specific OCMBs; **Mo** = months; **MS** = Multiple Sclerosis; **NFL** = Neurofilament; **NR** = Not reported; **OCGBs** = Oligoclonal IgG Bands; **OCMBs** = Oligoclonal IgM Bands; **OR** = Odd Ratio; **pRNFL** = peripapillary Retinal Nerve Fiber Layer; **PPMS** = Primary Progressive MS; **RR** = Relative Risk; **RRMS** = Relapsing-Remitting MS; **SPMS** = Secondary Progressive MS; **WBC** = white blood cell; **Yr** = year;

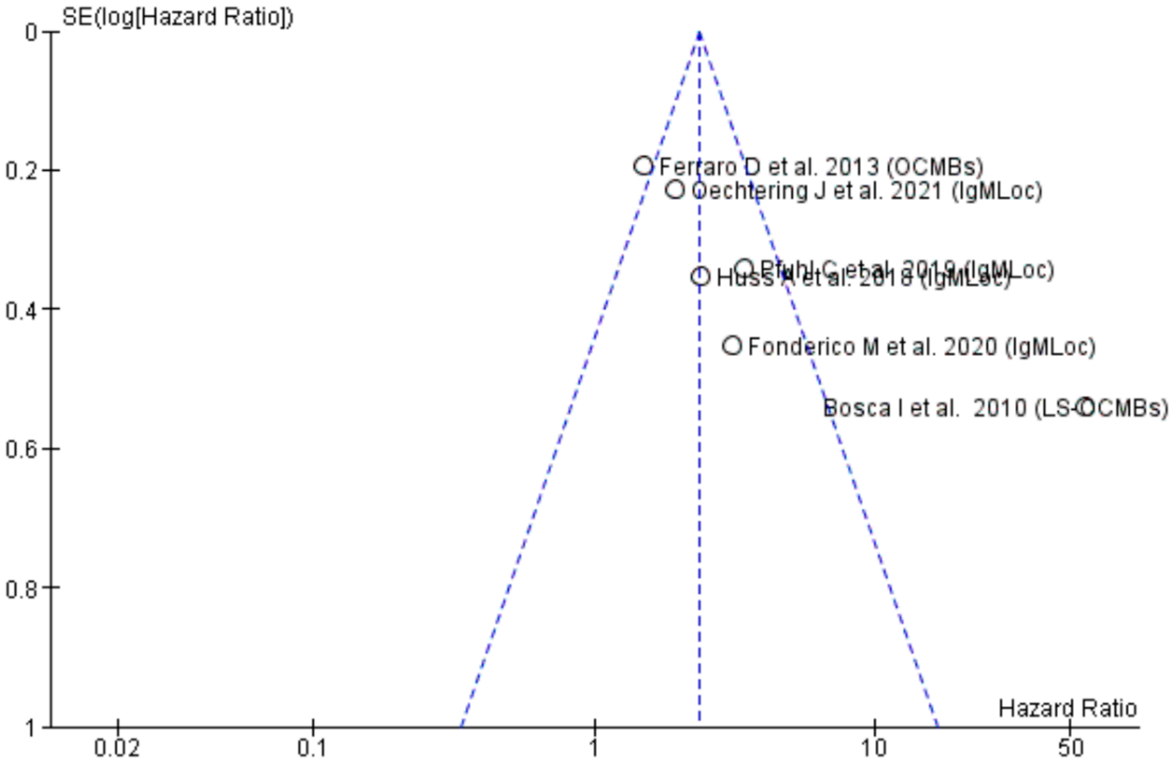

Figure S1. Forest plot for the risk of a second clinical relapse. Only studies with the same method of intrathecal IgM detection are analyzed.
